# Automated Evaluation of Ellipsoid Zone At-Risk Burden for Detection of Hydroxychloroquine Retinopathy

**DOI:** 10.3390/jpm14050448

**Published:** 2024-04-25

**Authors:** Katherine E. Talcott, Gagan Kalra, Hasan Cetin, Yavuz Cakir, Jon Whitney, Jordan Budrevich, Jamie L. Reese, Sunil K. Srivastava, Justis P. Ehlers

**Affiliations:** 1 The Tony and Leona Campane Center for Excellence in Image-Guided Surgery and Advanced Imaging Research, Cole Eye Institute, Cleveland Clinic, Cleveland, OH 44195, USAcetinh@ccf.org (H.C.); budrevj@ccf.org (J.B.);; 2 Cole Eye Institute, Cleveland Clinic Foundation, Cleveland, OH 44195, USA

**Keywords:** hydroxychloroquine retinopathy, ellipsoid zone integrity, quantitative optical coherence tomography, automated feature segmentation

## Abstract

Background: Screening for hydroxychloroquine (HCQ) retinopathy is crucial to detecting early disease. A novel machine-learning-based optical coherence tomography (OCT) biomarker, Ellipsoid Zone (EZ) At-Risk, can quantitatively measure EZ alterations and at-risk areas for progressive EZ loss in a fully automated fashion. The purpose of this analysis was to compare the EZ At-Risk burden in eyes with HCQ toxicity to eyes without toxicity. Methods: IRB-approved image analysis study of 83 subjects on HCQ and 44 age-matched normal subjects. SD-OCT images were reviewed for evidence of HCQ retinopathy. A ML-based, fully automatic measurement of the percentage of the macular area with EZ At-Risk was performed. Results: The mean age for HCQ subjects was 67.1 ± 13.2 years and 64.2 ± 14.3 years for normal subjects. The mean EZ At-Risk macular burden in the “toxic” group (n = 38) was significantly higher (10.7%) compared to the “non-toxic” group (n = 45; 2.2%; *p* = 0.023) and the “normal” group (1.4%; *p* = 0.012). Additionally, the amount of EZ At-Risk burden was significantly correlated with the HCQ dose based on the actual (*p* = 0.016) and ideal body weight (*p* = 0.033). Conclusions: The novel biomarker EZ-At Risk was significantly higher in subjects with evidence of HCQ retinopathy as well as significantly associated with HCQ dose. This novel biomarker should be further evaluated as a potential screening tool for subjects on HCQ.

## 1. Introduction

Hydroxychloroquine (HCQ) is a mainstay of treatment for connective tissue disorders but can cause irreversible retinal toxicity and permanent and progressive vision loss, even after medication cessation [1,2,3,4,5]. Risk factors for retinopathy include an excessive daily dose, cumulative dose, duration of treatment, and concurrent macular disease [4,6,7,8,9,10,11,12,13]. Primary screening tests include spectral domain optical coherence tomography (OCT), automated visual fields (VFs), and fundus auto-fluorescence (FAF). OCT is often the frontline testing performed and can detect the classic features of HCQ retinopathy [4,10,14,15]. These changes include loss of the parafoveal ellipsoid zone (EZ), parafoveal thinning of the outer nuclear layer (ONL) and inner plexiform layer (IPL), the “flying saucer” sign, and peripapillary nerve fiber layer thinning [7,16,17,18]. Groups have analyzed the OCTs of subjects with known HCQ retinopathy to retrospectively identify findings on OCT that may precede the classic macular changes [19]. One report found that parafoveal ONL thinning, disruption of the parafoveal interdigitation zone, and reduced reflectivity of the parafoveal EZ may precede disruption of the parafoveal EZ [7]. Another study described that loss of a clear continuous interdigitation zone was an early OCT change of HCQ retinopathy in subjects with otherwise normal screening metrics that was visible before subjects progressed to advanced parafoveal outer retinal disruption and/or paracentral visual field defects [20]. Other groups have recently identified OCT retinal thickness deviation maps and rapid macular thinning as biomarkers of retinopathy [21,22].

Loss of ellipsoid zone (EZ) integrity on OCT is a hallmark feature of HCQ retinopathy, but early alterations can be subtle [16,23,24,25]. The availability of an automated platform for enhanced OCT assessment, including quantitatively analyzing outer retinal metrics with the opportunity for a visual representation of these EZ-retinal pigment epithelium (RPE) thickness maps, could help facilitate the earlier detection of subclinical toxicity, including for eye care providers who are not retina specialists [16,23,24]. An automated deep-learning (DL)-enhanced EZ mapping tool with EZ-RPE segmentation that allows for line-by-line verification has been developed to better quantify and understand macular disease and has been linked to outcomes and disease severity in numerous retinal disorders [26,27,28,29]. This mapping tool has been used to examine subjects with HCQ retinopathy. Significant reductions in outer retinal parameters were found in these eyes, including partial EZ attenuation that was parafoveal in more mild disease and more diffuse with worse disease [25]. Additionally, machine learning algorithms utilizing clinical history and advanced OCT segmentation from eyes on HCQ have been developed to both detect and predict HCQ retinopathy [30].

Novel, automated imaging biomarkers have been developed to further evaluate EZ. EZ At-Risk is a fully automated deep-learning OCT biomarker defined by regions of unhealthy EZ but excluding regions of atrophy [31]. The initial focus and target of this biomarker was in nonexudative age-related macular degeneration [31].

Given that HCQ retinopathy is characterized by loss of EZ integrity, this study was initiated to explore whether EZ At-Risk could be used to identify HCQ retinopathy. A fully automated biomarker that can detect these subtle changes could be a potential screening endpoint for subjects on HCQ. The purpose of this study is to evaluate differences in EZ At-Risk between subjects with HCQ toxicity, HCQ subjects without toxicity, and normal controls.

## 2. Materials and Methods

This was an institutional review-board-approved retrospective image analysis study and complied with the declarations of the tenets of Helsinki. The requirement for informed consent was waived by the institutional review board due to the retrospective nature of this analysis. Thus, written informed consent was not obtained for this analysis.

### 2.1. Study Subjects and Data Collection

The study utilized clinical and OCT imaging data from subjects on HCQ therapy and age-matched healthy controls. Clinical data included age, gender, ethnicity, height, weight, daily HCQ dose, HCQ dose in milligram (mg)/kilogram (kg) actual body weight, HCQ dose in mg/kg ideal body weight, duration on HCQ therapy, cumulative dose of HCQ, systemic autoimmune disease status, co-existing kidney disease, concurrent tamoxifen use, and visual acuity. All subjects in this analysis underwent spectral-domain (SD)-OCT imaging using the macular cube protocol (512 × 128 A-scans) with the Cirrus HD-OCT (Zeiss, Oberkochen, Germany) covering a 6 × 6 mm fovea-centered area. SD-OCT images was collected for all subjects (one eye per subject) and reviewed by two retina specialists (KET, JPE) for evidence of HCQ retinopathy in a masked manner. Additionally, any subjects with concern for co-existing macular disease were excluded. Subjects on HCQ with retinopathy were labelled as “toxic”, and the remaining subjects on HCQ were labelled as “non-toxic”. The subjects in the “non-toxic” and “normal” groups included in this analysis were chosen at random to match the ”toxic” subjects in a relative 1:1 match largely based on age [26,30]. Unfortunately, subjects were unable to be matched based on baseline clinical characteristics, given the number involved.

### 2.2. Automatic Ellipsoid Zone At-Risk Quantification

A previously described deep learning model trained to automatically identify and quantify regions of attenuated EZ (based on ground truth segmentation masks consisting of an EZ-RPE thickness of <10-micron) was utilized to analyze the OCT images [31]. The training methodology of this model has been previously described. In brief, training masks were generated from regions with an EZ-RPE thickness of 10 microns or less in eyes with nonexudative age-related macular degeneration. Subsequently, a modified U-Net architecture was deployed for model training, and a fully automatic segmentation and measurement of regions with EZ At-Risk was achieved.

### 2.3. Statistical Analysis

All statistical analyses were performed using R (v4.0.1, Bell Laboratories, Murray Hill, NJ, USA). Matching between healthy controls and subjects on HCQ therapy was assessed by analyzing descriptive clinical and demographic statistics from these groups. The mean percentage area of EZ-At-Risk was compared between the “toxic”, “non-toxic”, and “normal” groups. Normalcy of data was assessed with a Shapiro–Wilk test. Group means were compared using an ANOVA test with a Tukey’s post-hoc test for a normal distribution of data and Kruskal–Wallis test with Dunn’s post-hoc test for a non-normal data distribution. Pearson’s correlation was utilized to identify associations of EZ At-Risk with clinical parameters for normal distributions, and Spearman’s correlation was utilized for non-parametric calculations. Statistical significance was inferred at *p* < 0.05.

## 3. Results

A total of 83 eyes from 83 subjects on HCQ, including 38 that were determined to have evidence of HCQ retinopathy based on OCT review, and 44 eyes from 44 age-matched normal subjects were included in the analysis. The baseline clinical characteristics are shown in Table 1. The mean age was 67.1 ± 13.2 years in the HCQ cohort and 64.2 ± 14.3 years in the normal eyes. In the HCQ cohort, the majority of subjects were female (n = 73; 88%) and Caucasian (n = 65; 78%). Baseline visual acuity was a mean of 20/25 (logMAR 0.2 ± 0.3). The mean daily HCQ dose was 380.7 ± 64.4 mg, which corresponded to a mean actual body weight dose of 5.3 ± 1.6 mg/kg and mean ideal body weight dose of 6.9 ± 1.4 mg/kg. Rheumatoid arthritis (n = 37; 45%) and lupus (n = 31; 37%) were the most common clinical indications for HCQ use, and risk factors for HCQ toxicity, including concurrent tamoxifen use (n = 2; 2%) and kidney disease (n = 6; 7%), were relatively rare.

**Figure 1:**
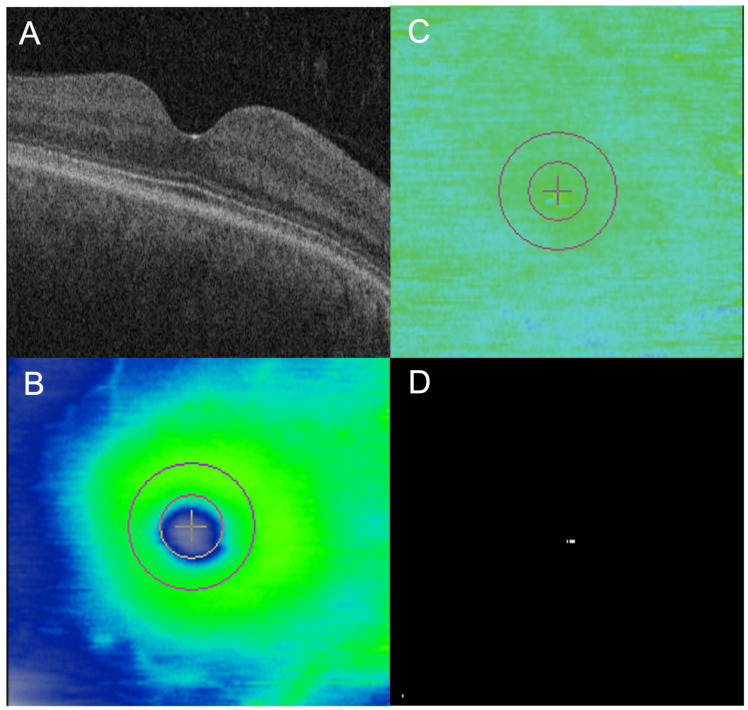
Case example of ellipsoid zone (EZ) at-risk in non-toxic eye while on hydroxychloroquine (HCQ). A 62 year old woman on HCQ; daily dosing, based on actual body weight, was 5.65 mg/kg for 7 years at the time of the OCT (**A**,**B**). There was no significant partial parafoveal ellipsoid zone (EZ)-retinal pigment epithelium (RPE) attenuation on en face EZ-RPE mapping (**C**), which would have appeared as areas of purple, or EZ At-Risk on en face mapping (**D**), which would have appeared as areas of white.

**Figure 2:**
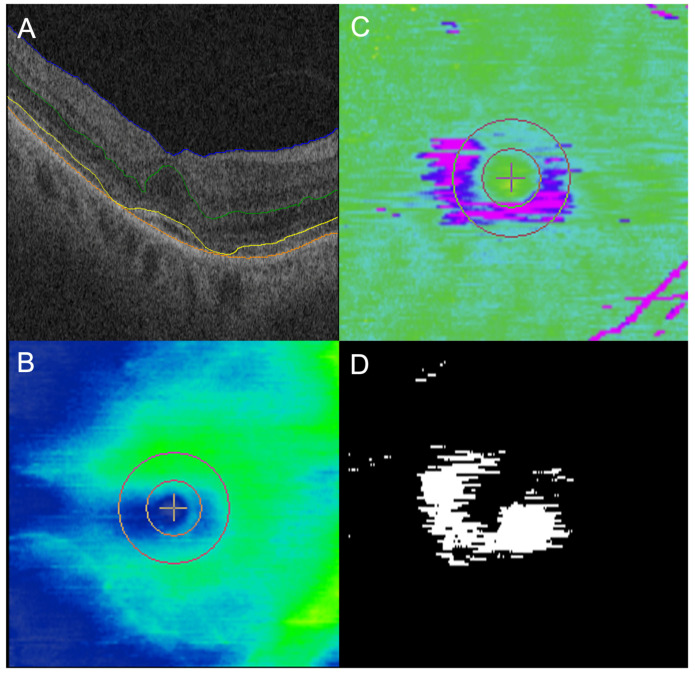
Case example of significant parafoveal ellipsoid zone (EZ) at-risk in eye in toxic eye while on hydroxychloroquine (HCQ). A 70 year old woman on HCQ; daily dosing, based on actual body weight, was 5.22 mg/kg for 6 years at the time of the OCT (**A**,**B**). There were significant areas of parafoveal partial ellipsoid zone (EZ)-retinal pigment epithelium (RPE) attenuation on en face EZ-RPE mapping (**C**), which appear as purple. These areas correlated well with the white areas of EZ At-Risk on en face mapping (**D**).

**Figure 3:**
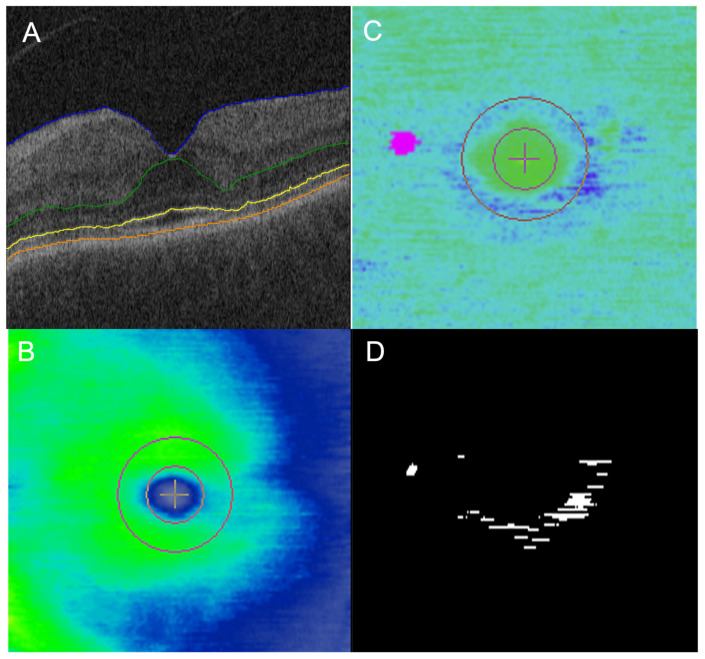
Case example of parafoveal ellipsoid zone (EZ) at-risk in eye in toxic eye while on hydroxychloroquine (HCQ). A 71 year old man on HCQ; daily dosing, based on actual body weight, was 5.45 mg/kg for 10 years at the time of the OCT (**A**,**B**). There were subtle areas of parafoveal partial ellipsoid zone (EZ)-retinal pigment epithelium (RPE) attenuation on en face EZ-RPE mapping (**C**), which appear as purple. These areas correlated well with the white areas of EZ At-Risk on en face mapping (**D**).

The mean EZ At-Risk macular burden in the “toxic” group was significantly higher (10.7%) compared to the “non-toxic” group (2.2%; *p* = 0.023). The mean EZ At-Risk in the “toxic” group was also significantly higher than the “normal” group (1.4%; *p* = 0.012). There was no significant difference between the “non-toxic” and “normal” group (*p* = 0.580; Table 2). Eyes in the “non-toxic” group generally did not show any significant areas of EZ At-Risk, with a representative subject shown in Figure 1. In contrast, eyes in the “toxic” group showed parafoveal areas of EZ At-Risk that corresponded to areas of partial EZ-RPE attenuation on en face mapping in the representative subject (Figure 2 and Figure 3).

Additionally, the percentage area of EZ At-Risk was compared to clinical parameters in subjects on HCQ in both the “toxic” and “non-toxic” groups. The percentage area of EZ At-Risk was significantly correlated with the HCQ dose based on the actual (*p* = 0.016) and ideal body weight (*p* = 0.033) and trended towards a significant correlation with the cumulative dose (*p* = 0.069). Subject age, vision, HCQ daily dose, and duration of HCQ therapy were not significantly correlated with EZ At-Risk (Table 3; all *p* > 0.136).

## 4. Discussion

In this study, a novel fully automated OCT biomarker examining EZ At-Risk was examined in subjects on HCQ and with evidence of HCQ retinopathy. Loss of EZ integrity on OCT is a classic feature of HCQ toxicity, but early alterations can be subtle, even for those well-versed in interpreting OCTs. A fully automated metric able to detect EZ attenuation may provide an opportunity for screening and improved detection of HCQ retinopathy. This assessment found significantly higher levels of EZ At-Risk in subjects with HCQ retinopathy (10.7%) as compared to subjects on HCQ without evidence of retinopathy (2.2%), a nearly five-fold increase. The macular EZ At-Risk burden was also found to be significantly correlated with HCQ dose based on actual and ideal body weight. There was no significant difference in EZ At-Risk between HCQ subjects without retinopathy and age-matched controls. While outer retinal metrics on OCT have previously been noted in subjects with HCQ retinopathy, the application of this novel and fully automated biomarker is unique [7,23,25,30,32].

This study builds on previous work demonstrating the utility of using an automated EZ mapping platform to identify outer retinal changes on OCT in HCQ subjects [25,30]. This EZ mapping tool was previously utilized in eyes with clinically recognized HCQ retinopathy, finding a significant reduction of outer retinal parameters, including en face EZ attenuation, compared to controls. The current analysis demonstrated similar results, specifically a significant increase in EZ At-Risk in subjects with HCQ retinopathy as compared to HCQ retinopathy subjects and age-matched controls. The pattern of EZ At-Risk on en face mapping for eyes in the “toxic” group showed a similar pattern to those with mild toxicity described in previous reports, namely parafoveal attenuation, which would be expected for HCQ retinopathy [25]. This study also helps validate this novel EZ biomarker and reinforces its generalizability beyond nonexudative age-related macular degeneration for a unique indication and points toward its promise as an OCT biomarker and screening tool [31].

Given the progressive and irreversible nature of HCQ retinopathy, early detection of toxic changes on OCT is of great interest. For instance, Lally et al. examined 30 eyes with HCQ retinopathy and found that parafoveal ONL thinning, disruption of the parafoveal interdigitation zone, and reduced reflectivity of the parafoveal EZ preceded parafoveal EZ disruption [7]. Disruption of the parafoveal interdigitation zone is likely reflected in the measurement of EZ At-Risk. Additionally, Garrity et al. reported on 10 subjects with HCQ retinopathy with OCT changes but reassuring visual field tests, finding early OCT alterations including parafoveal EZ attenuation [20]. These studies describe subtle qualitative OCT that can be easily missed. This is true not only for retinal specialists who are well-versed in OCTs, but also for other eye care providers, including optometrists and comprehensive ophthalmologists, who may be less adept at detecting subtle outer retinal changes on OCT. This highlights the need for better OCT screening tools and fully automated biomarkers of HCQ retinopathy.

Prior studies have shown that HCQ subjects have stable OCTs until they may develop retinopathy rather than a slow accumulation of toxic changes [23]. This study supports this, given that there was no significant difference in EZ At-Risk between the “non-toxic” and “normal” group. However, the relatively small size of the groups may not be able to detect a difference.

There are several limitations to this study, largely resulting from its retrospective nature and relatively small size. OCT orientation was not prospectively optimized. Significant tilt may result in alterations to EZ reflectivity, and it is not clear of this impact on EZ At-Risk detection. Additionally, other tests and imaging modalities, including VFs, FAF, and multifocal electroretinogram, may have been used clinically for HCQ screening, but were not included in this study and should be investigated to correlate for changes in the future. Additionally, the relatively small sample size may have limited additional correlations of EZ At-Risk with clinical characteristics. The sample size also limited the ability to perform matching based on clinical characteristics, including HCQ dose and duration, although there were no significant differences between the HCQ cohorts. Although this OCT biomarker was found to be significantly associated with HCQ dose based on ideal and actual body weight, the sample size might not have been large enough to detect a difference for the other clinical characteristics. Finally, EZ At-Risk was not compared to other quantitative outer retinal metrics, and examined these metrics at a single timepoint rather than dynamically.

Overall, this study examines a novel fully automated OCT biomarker, examining EZ At-Risk in HCQ subjects. The assessment demonstrated increased EZ At-Risk in HCQ subjects with retinopathy compared to HCQ subjects without retinopathy as well as age-matched controls. This metric was also significant associated with HCQ dose based on actual and ideal body weight. Our findings provide a “proof of concept” that this biomarker has a significant potential as a screening tool, especially given that it is fully automated. Further studies are needed to better elucidate this novel biomarker longitudinally in HCQ subjects, compare to other quantitative metrics, evaluate for potential threshold risks for toxicity classification, and better correlate with disease severity.

## Figures and Tables

**Table 1:** Demographics and baseline clinical characteristics of included subjects and eyes.

Parameter	Normal Group (n = 44 Eyes)	Total HCQ Cohort (n = 83 Eyes)	Toxic HCQ Group (n = 38 Eyes)	Nontoxic HCQ Group (n = 45 Eyes)	*p*-Value
Age (years; mean ± SD)	64.2 ± 14.3	67.1 ± 13.2	69.5 ± 13.6	65.1 ± 12.6	0.128
Gender (n)					
Male	19	10	3	7	
Female	25	73	35	38	
Ethnicity (n)					
White	28	65	28	37	
Black	12	15	8	7	
Hispanic	3	1	1	0	
Asian	0	2	1	1	
Others	2	0	0	0	
Declined to answer	2	0	0	0	
Daily HCQ Dose (mg; mean ± SD)	-	380.7 ± 64.4	378.9 ± 57.7	382.2 ± 68.4	0.816
HCQ daily dose per ideal body weight (mg/kg; mean ± SD)	-	6.9 ± 1.4	7.2 ± 1.5	6.7 ± 1.3	0.155
HCQ daily dose per actual body weight (mg/kg; mean ± SD)	-	5.3 ± 1.6	5.6 ± 1.8	5.1 ± 1.4	0.122
Duration on HCQ therapy (years; mean ± SD)	-	10.9 ± 4.2	10.9 ± 5.1	10.8 ± 3.3	0.810
Cumulative HCQ dose (grams; mean ± SD)	-	1505.8 ± 633.2	1520.1 ± 765.3	1493.7 ± 504.3	0.851
Systemic autoimmune illness (n)					
Rheumatoid arthritis	-	37	14	23	
Lupus	-	31	15	16	
Other	-	15	9	6	
Concurrent kidney disease (n)	-	6	2	4	
Concurrent tamoxifen use (n)	-	2	0	2	
Visual Acuity (Snellen logMAR; mean ± SD)	0.0 ± 0.2	0.2 ± 0.3	0.2 ± 0.3	0.2 ± 0.3	0.479

SD = standard deviation, cm = centimeter, kg = kilogram, HCQ = hydroxychloroquine, mg = milligram; *p*-value bolded if *p* < 0.05.

**Table 2:** Comparison of mean percentage area of EZ At-Risk between various groups.

Mean Macular EZ At-Risk Burden (in %)		
Toxic group	Nontoxic group	*p*-value
10.7 ± 23.3	2.2 ± 5.4	**0.023**
Toxic group	Normal group	*p*-value
10.7 ± 23.3	1.38 ± 5.72	**0.012**
Nontoxic group	Normal group	*p*-value
2.2 ± 5.4	1.38 ± 5.72	0.580

Compared using Kruskal–Wallis test and Dunn’s post-hoc test; bolded if *p* < 0.05.

**Table 3:** Correlation of percentage area of EZ At Risk in subjects on hydroxychloroquine (HCQ) therapy with various clinical parameters.

Parameter	R	*p*-Value
Cumulative HCQ dose	0.20	0.069
HCQ daily dose per kg of ideal body weight	0.23	**0.033**
HCQ daily dose per kg of actual body weight	0.26	**0.016**
Duration on HCQ	0.17	0.136
Age	−0.10	0.362
Daily HCQ Dose	0.10	0.369
Snellen VA	−0.05	0.675
Snellen logMAR VA	0.02	0.868

Spearman’s correlation; bolded if *p* < 0.05; HCQ = hydroxychloroquine, kg = kilogram, VA = visual acuity.

## Data Availability

Data are contained within the article.
